# Research Advances of Cellular Nanoparticles as Multiplex Countermeasures

**DOI:** 10.1021/acsnano.4c09830

**Published:** 2024-10-23

**Authors:** Jiayuan Alex Zhang, Kailin Feng, Wei-Ting Shen, Weiwei Gao, Liangfang Zhang

**Affiliations:** Aiiso Yufeng Li Family Department of Chemical and Nano Engineering, Shu and K.C. Chien and Peter Farrell Collaboratory, University of California San Diego, La Jolla, California 92093, United States

**Keywords:** nanomedicine, cellular nanoparticle, cellular nanosponge, cell membrane, detoxification, multiplex neutralization, continuous neutralization, enzyme encapsulation, membrane modification

## Abstract

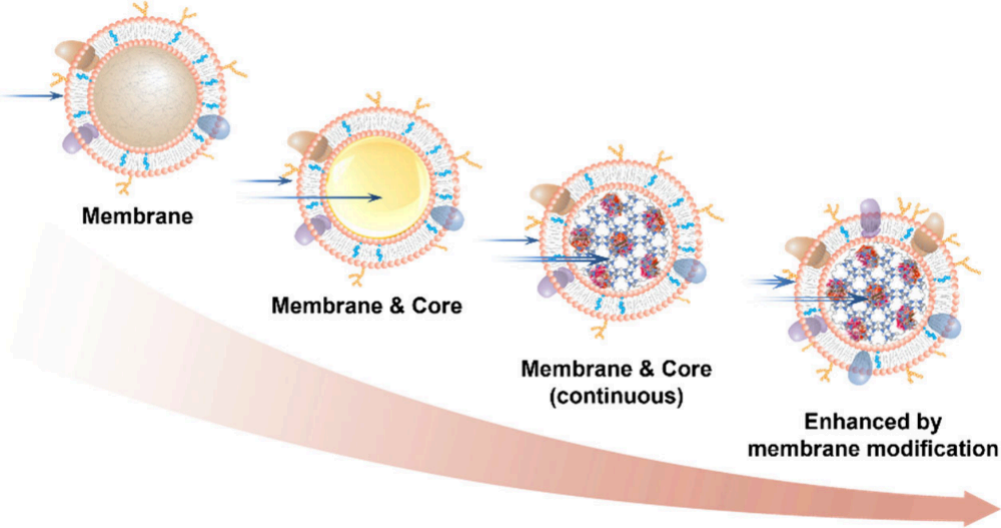

Cellular nanoparticles (CNPs), fabricated by coating natural cell membranes onto nanoparticle cores, have been widely used to replicate cellular functions for various therapeutic applications. Specifically, CNPs act as cell decoys, binding harmful molecules or infectious pathogens and neutralizing their bioactivity. This neutralization strategy leverages the target’s functional properties rather than its structure, resulting in broad-spectrum efficacy. Since their inception, CNP platforms have undergone significant advancements to enhance their neutralizing capabilities and efficiency. This review traces the research advances of CNP technology as multiplex countermeasures across four categories with progressive functions: neutralization through cell membrane binding, simultaneous neutralization using both cell membrane and nanoparticle core, continuous neutralization via enzymatic degradation, and enhanced neutralization through membrane modification. The review highlights the structure–property relationship in CNP designs, showing the functional advances of each category of CNP. By providing an overview of CNPs in multiplex neutralization of a wide range of chemical and biological threat agents, this article aims to inspire the development of more advanced CNP nanoformulations and uncover innovative applications to address unresolved medical challenges.

The pursuit of enhancing therapeutic nanoparticle performance has led to innovative designs with distinct structures and intricate biointerfacing capabilities.^1,2^ Among these advancements, cell membrane-coated nanoparticles, created by coating natural cell membranes onto synthetic substrates, have attracted much attention.^3^ This approach bestows nanoparticles with cell-like functionalities that are difficult to replicate through traditional methods, while the synthetic substrates provide engineering flexibility and versatility. These nanoparticles are referred to as “cellular nanoparticles” or “CNPs” to highlight their biomimicry of natural cells. Initially, researchers coated red blood cell (RBC) membrane onto polymeric nanoparticle cores to mimic the long-circulation property of natural RBCs, leading to RBC membrane-coated nanoparticles (denoted “RBC-CNPs”) with prolonged residence time in vivo.^4^ This landmark study quickly inspired the concept of using CNPs as decoys to neutralize pore-forming toxins (PFTs) by mimicking susceptible RBCs.^5^

All pathological agents must interact with host cells to exert their bioactivity. Leveraging this principle, researchers develop neutralizing CNPs by selecting appropriate cell membranes from the top down, rather than deciphering the structure of the targets to create specific agents from the bottom up.^6^ This shift from identifying causative agents to mimicking host cells has become the defining feature of CNPs for multiplex neutralization of numerous agents, unlocking exciting possibilities for function-driven, broad-spectrum neutralization solutions. Utilizing natural cell membranes enables researchers to leverage complex cellular functions that are difficult to replicate through bottom-up synthesis. Meanwhile, synthetic substrates provide structural stability and engineering flexibility. These substrates, made from various materials, can also enhance neutralization by encapsulating bioactive molecules or directly absorbing harmful agents. Following the initial development of RBC-CNPs, researchers have expanded their use beyond neutralizing PFTs to intercept harmful agents such as pathological antibodies and nerve agents.^7,8^ As membranes from various cell types have been successfully coated onto different nanoparticle materials, CNPs have been applied to neutralize a broader range of targets, including bacteria, viruses, neurotoxins, and factors involved in inflammatory disorders.^9−11^ Concurrently, robust methods have been developed to enhance the functionality of the cell membranes on CNPs, further improving the effectiveness of neutralization.^12^

Throughout their development, CNP designs have advanced continuously and rapidly. To date, four categories of CNPs have been developed as multiplex countermeasures against a wide range of chemical and biological threat agents. These categories are distinguished by how the membrane shell and the nanoparticle core interact with the target agents to achieve effective neutralization (Figure 1). In the first category, CNPs rely solely on the membrane shell to bind with pathological agents for neutralization, while the nanoparticle cores primarily stabilize the CNP structure. The second category introduces cores that bind with agents permeating the membrane, enhancing neutralization through mechanisms such as physical dissolution or binding via encapsulated binding moieties. The third category of CNPs encapsulates enzymes capable of degrading agents for continuous neutralization, overcoming the solubility and binding stoichiometry limitations of the previous category. In the fourth category, the cell membranes are further modified to increase neutralization efficacy by increasing the density of binding moieties, prolonging CNP residence time for more efficient neutralization, or enhancing membrane permeability to boost enzyme degradation.

**Figure 1:**
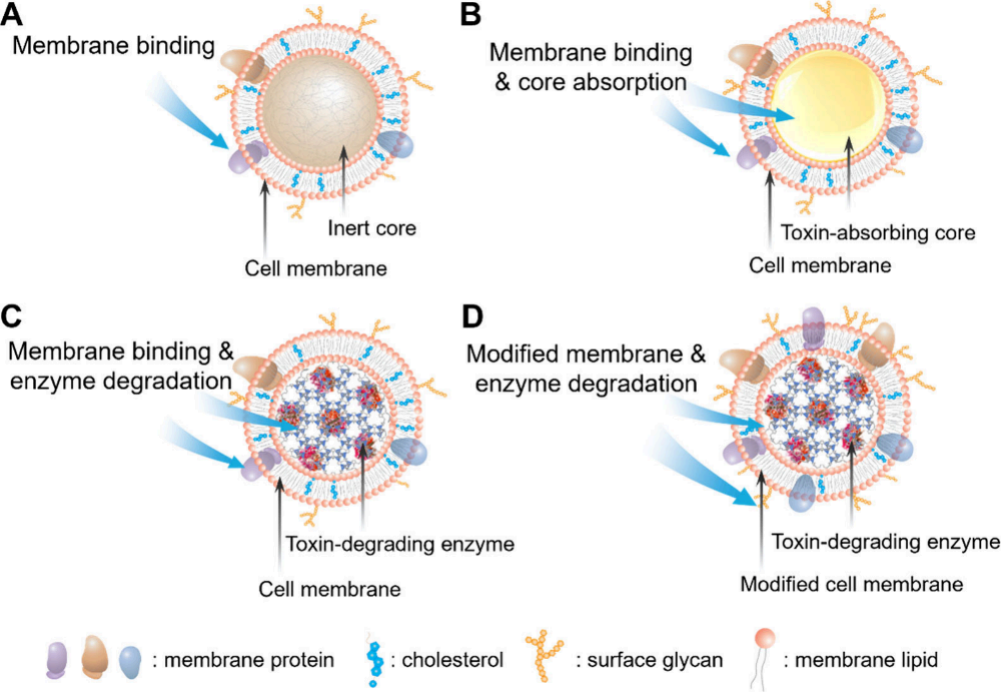
Schematic representations of the four categories of cellular nanoparticle (CNP) designs with progressive functions for multiplex neutralization. (A) The first category of CNPs utilizes the cell membrane shell for binding with pathological agents, while the nanoparticle core provides structural stability. (B) The second category of CNPs features cores that can bind with agents permeating the membrane, enhancing neutralization efficacy. (C) The third category of CNPs encapsulates enzymes capable of degrading agents, allowing for continuous neutralization. (D) The fourth category of CNPs is constructed with modified cell membranes to improve neutralization efficiency.

This article reviews representative designs of CNPs across four categories, emphasizing their distinct mechanisms of neutralization by highlighting their underlying structure–property relationships. By reviewing examples from the development of each CNP category, we aim to provide a comprehensive understanding of the progress in CNP technology, with the hope of inspiring additional applications and advancements in therapeutic interventions.

## Neutralization via Cell Membrane

**Figure 2:**
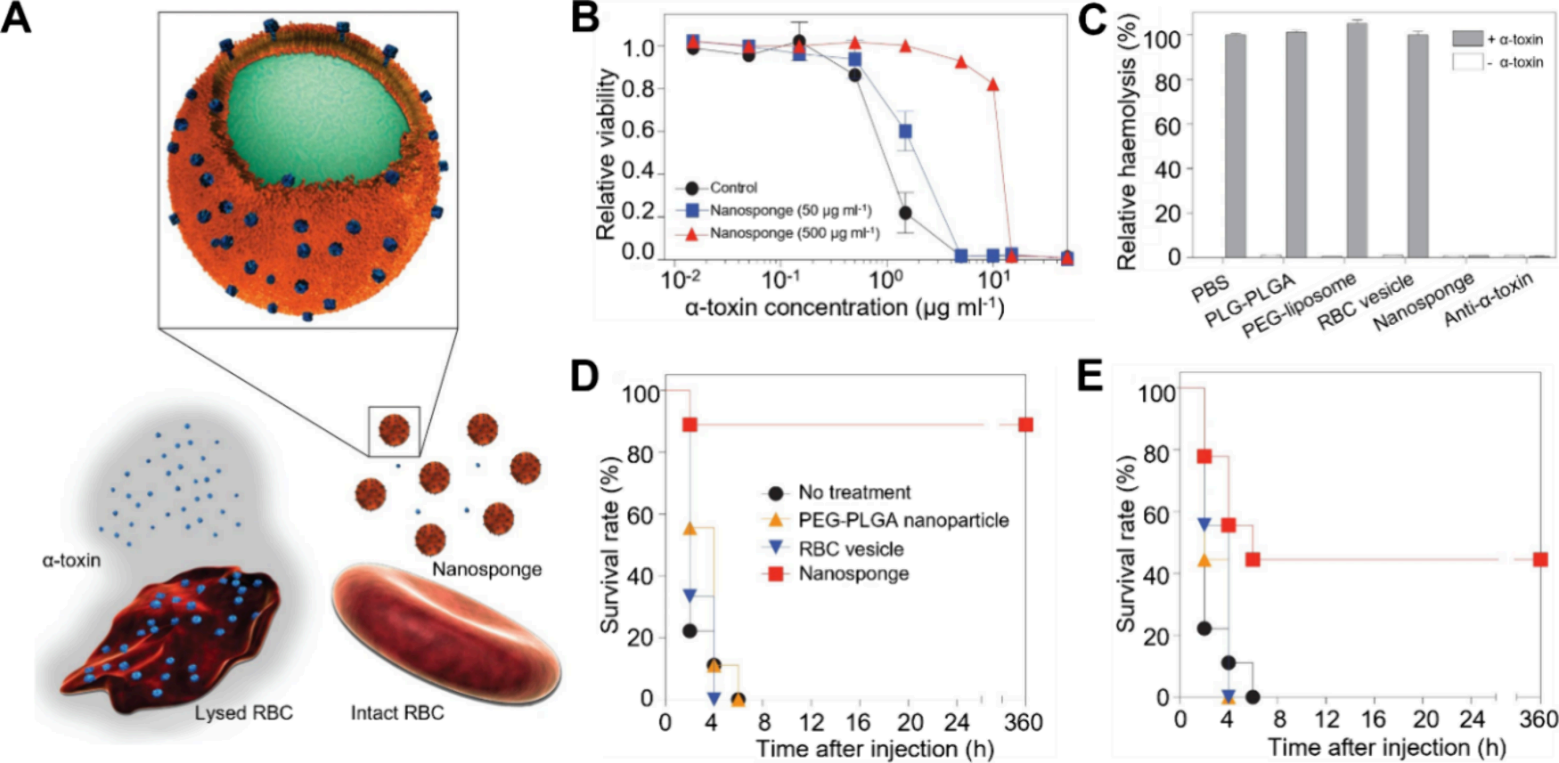
CNPs made with red blood cell membrane (RBC-CNPs, denoted as “RBC-NPs” in the original study, ref (5)) were used to neutralize pore-forming toxins (PFTs). (A) Schematic representation of RBC-NPs and their mechanism for neutralizing PFTs. These nanoparticles were also named “nanosponges” to emphasize their working mechanisms of “soaking up” harmful toxins for neutralization. (B) Dose-dependent neutralization of α-toxin by RBC-NPs against human umbilical vein endothelial cells (HUVECs). Error bars represent standard deviations (*n* = 6). (C) Quantification of hemolysis with anti-α-toxin as a positive control and polyethylene glycol (PEG)-modified poly(lactic-*co*-glycolic acid) nanoparticles (PEG-PLGA) or PEG-functionalized liposomes (PEG-liposome) as negative controls. Error bars represent standard deviations (*n* = 3). (D and E) Survival rates of mice over 15 days following intravenous injection of α-toxin (75 μg/kg). Mice received 80 mg/kg of RBC-NP nanosponges, RBC vesicles, or PEG-PLGA nanoparticles intravenously 2 min either before (D) or after (E) toxin injection (*n* = 9). Adapted with permission from ref (5). Copyright 2013 Springer Nature Limited.

The potential of CNPs for multiplex neutralization was first demonstrated through the neutralization of pore-forming toxins (PFTs). These toxins compromise cellular membrane integrity by creating pores. PFTs constitute the largest class of bacterial toxins, and their diverse molecular structures and epitopic targets pose significant challenges for effective neutralization.^13^ However, RBC–CNPs exploit the natural affinity between the cell membrane and the toxins, effectively absorbing and neutralizing the toxins regardless of their structure. RBC–CNPs have successfully neutralized α-hemolysin from methicillin-resistant *Staphylococcus aureus* (MRSA), protecting mice from toxin-induced lethality (Figure 2).^5^ In these studies, CNPs were named “nanosponges” to emphasize their mechanisms of “soaking up” harmful toxins for neutralization. Subsequently, RBC–CNPs have demonstrated the ability to neutralize other types of PFTs, including melittin, streptolysin O from Group A Streptococcus, listeriolysin O from *Listeria monocytogenes*, and the entire secreted protein profile of MRSA, showcasing broad-spectrum, function-based bioneutralization.^14−18^ Additionally, RBC–CNPs have protected the retina in mouse endophthalmitis models by neutralizing PFTs from common intraocular infection-causing bacteria such as *Staphylococcus aureus*, *Enterococcus faecalis*, *Streptococcus pneumoniae*, and *Bacillus cereus*.^19,20^ Researchers have also embedded RBC–CNPs into hydrogels, creating composite materials or colloid gels for localized PFT neutralization.^21−23^

CNPs have also been utilized to neutralize other harmful agents. For instance, pathological antibodies are critical to type II immune hypersensitivity reactions, which destroy healthy tissues. Current treatments pose significant iatrogenic risks and often fail to achieve clinical remission for many patients.^24^ RBC-CNPs can function as decoys for pathological antibodies without drug-based immune suppression in this context.^7^ They have been shown to neutralize anti-RBC polyclonal IgG and preserve circulating RBCs in a mouse model of antibody-induced anemia. Similarly, immune thrombocytopenia purpura (ITP) is characterized by producing pathological autoantibodies that lower platelet counts.^25^ In this case, platelet membrane-coated nanoparticles have effectively neutralized antiplatelet antibodies and reduced bleeding time associated with platelet deficiency in a mouse model of antibody-induced thrombocytopenia.^26^

CNPs have also been employed to neutralize small molecular toxicants. For example, organophosphates (OPs), including lethal nerve agents, cause damage by irreversibly phosphorylating and inactivating acetylcholinesterase (AChE).^27^ Drawing inspiration from the natural expression of AChE on the RBC membrane, researchers utilized RBC-CNPs to scavenge OPs, successfully rescuing mice from OP-induced lethality.^8^ Additionally, mitochondrial outer membrane-coated CNPs have been used to neutralize toxic anticancer compounds such as ABT-263, offering a promising approach for treating drug overdose.^28^

**Figure 3:**
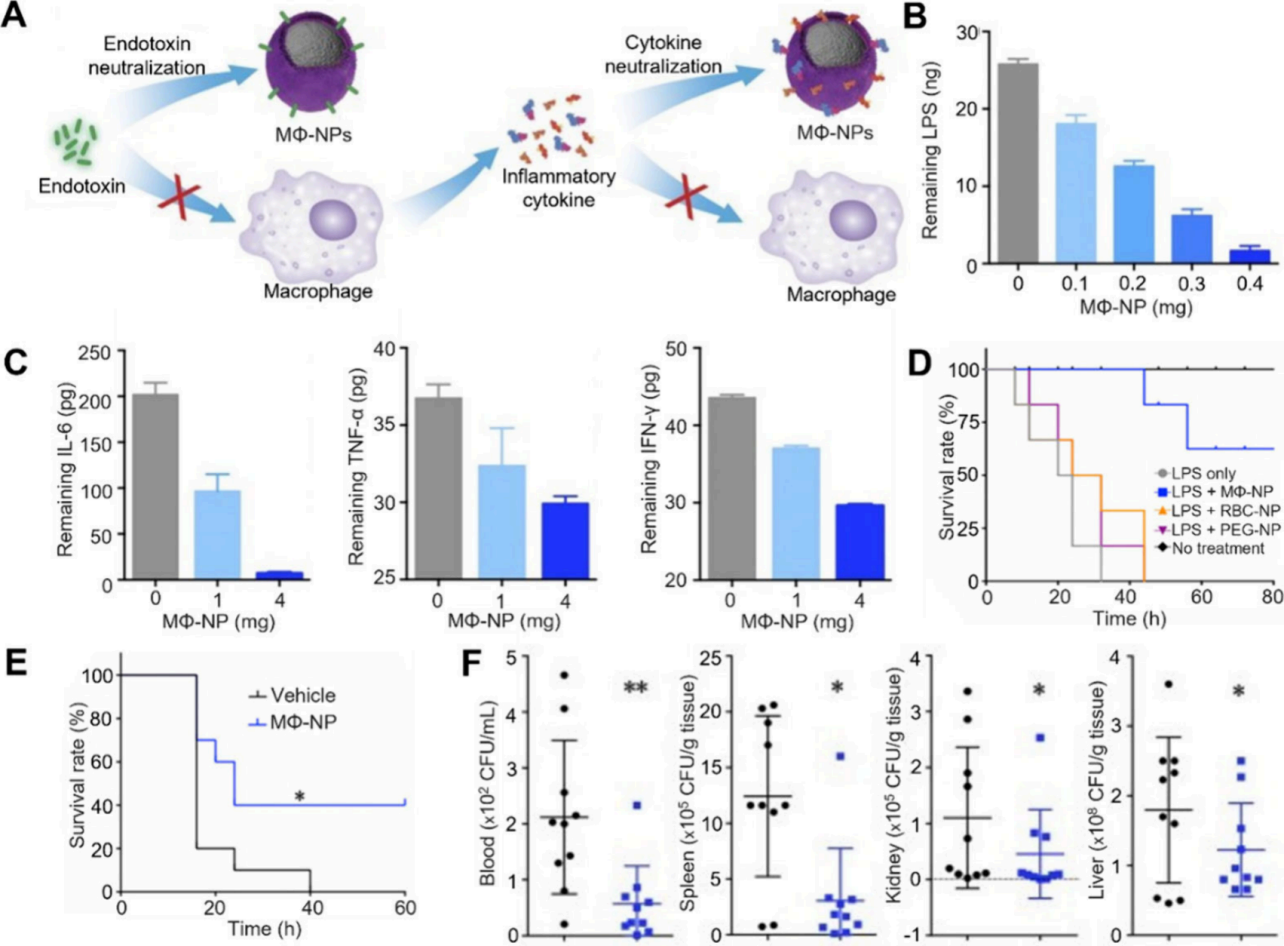
CNPs made with macrophage cell membrane (MΦ-CNPs, denoted as “MΦ-NPs” in the original study, ref (30)) were used to concurrently absorb endotoxins and proinflammatory cytokines for sepsis management. (A) Schematic representation illustrating the two-step process where MΦ-NPs neutralize endotoxin (LPS) and proinflammatory cytokines for managing sepsis. (B) Quantification of LPS removal using a fixed amount of LPS (25 ng) with varying amounts of MΦ-NPs. (C) In vitro removal of proinflammatory cytokines, including IL-6, TNF-α, and IFN-γ, using MΦ-NPs. (D) In vivo LPS neutralization by MΦ-NPs. Survival rates (*n* = 10) of mice injected with LPS alone or LPS mixed with MΦ-NPs, RBC-NPs, or PEG-functionalized PLGA nanoparticles (PEG-NPs). Untreated mice served as a control group. (E) In vivo therapeutic efficacy of MΦ-NPs evaluated using a mouse bacteremia model. Survival curve of bacteremic mice treated with MΦ-NPs (*n* = 10). (F) Bacterial counts in the blood, spleen, kidney, and liver at 4 h after intraperitoneal injection of MΦ-NPs. Adapted with permission from ref (29). Copyright 2017, the National Academy of Sciences of the United States of America.

Researchers have also developed CNPs to neutralize endotoxins (LPS) and inflammatory cytokines to manage inflammatory disorders better. In sepsis, which results from uncontrolled inflammatory responses to bacterial infections, LPS is a critical pathogenic trigger that produces proinflammatory cytokines.^30^ By using macrophage membrane to coat polymeric nanoparticles, the researchers demonstrated that the resulting MΦ-CNPs retain the antigenic exterior of the source cells, enabling them to concurrently bind effectively with LPS and inflammatory cytokines (Figure 3).^29^ It was shown that MΦ-CNPs reduced bacterial burden and prolonged survival in a mouse model of *Escherichia coli*-induced bacteremia. Researchers also developed neutrophil membrane-coated CNPs (Neutrophil-CNPs) for managing experimental rheumatoid arthritis.^10^ In this design, Neutrophil-CNPs mimicked host neutrophils, scavenging immunoregulatory molecules such as IL-1β and TNF-α responsible for sustaining inflammation and damaging the cartilage. Neutrophil-CNPs also penetrated deeper into the injured cartilage matrix for better chondroprotection. In a mouse model of collagen-induced arthritis and a human transgenic mouse model of arthritis, the Neutrophil-CNPs effectively reduced joint damage and suppressed overall arthritis severity.

**Figure 4:**
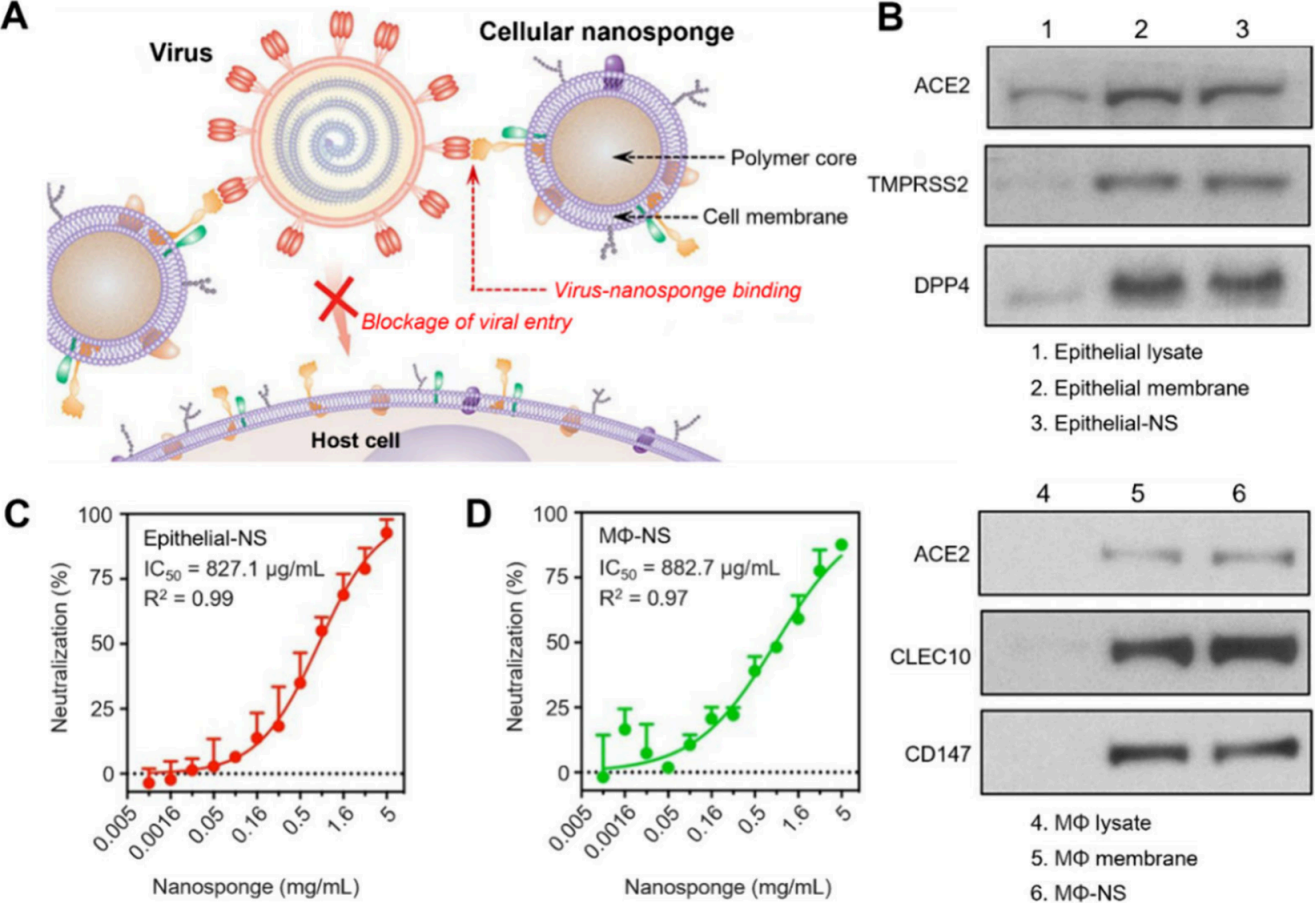
CNPs made with lung epithelia cell membrane or macrophage cell membrane (Epithelial-CNPs or MΦ-CNPs, denoted as “Epithelial-NS” or “MΦ-NS”, respectively, in the original study, ref (33)) were used to inhibit SARS-CoV-2 infectivity. (A) Schematic illustration of the mechanism of using CNPs to inhibit SARS-CoV-2 infectivity by blocking the viruses from entering the host cells. (B) Western blotting analysis revealing selective protein bands in cell lysate, cell membrane vesicles, and cellular nanosponges. (C and D) CNPs neutralize SARS-CoV-2 infectivity. The neutralization efficacy against SARS-CoV-2 infection by (C) Epithelial-NS and (D) MΦ-NS was evaluated using live SARS-CoV-2 viruses on Vero E6 cells. Each data set represents *n* = 3, with mean values presented as mean + standard deviation. Horizontal dashed lines indicate zero levels. Adapted with permission from ref (33). Copyright 2020, the American Chemical Society.

CNPs have also been developed to neutralize viruses and bacteria. For instance, CD4^+^ T cell membranes were coated onto polymeric nanoparticle cores to form T-cell CNPs. These CNPs inherit T cell surface receptors, including CD4, CCR5, and CXCR4 co-receptors, essential for HIV entry. By mimicking host T cells, these CNPs inhibited the viral infection of human peripheral mononuclear blood cells.^31^ Later, these T-cell CNPs were shown to neutralize a global panel of HIV isolates, including 125 HIV-1 pseudotyped viruses, demonstrating their broad and potent antiviral capabilities.^32^ CNPs coated with membranes from human lung epithelial type II cells (Epithelial-CNPs) or macrophages (MΦ-CNPs) displayed protein receptors such as ACE2 and CD147, which are crucial for the cellular entry of severe acute respiratory syndrome coronavirus 2 (SARS-CoV-2). These CNPs have shown dose-dependent efficacy in inhibiting the infectivity of SARS-CoV-2 viruses (Figure 4).^33^ Furthermore, gastric epithelial cell membranes were derived to coat onto nanoparticle cores for targeted binding to *Helicobacter pylori* for bacterial inhibition and antimicrobial delivery.^34^ In this example, another possible mechanism of action involves competitive binding, where CNPs made from *H. pylori* outer membrane vesicles competed with *H. pylori* bacteria for host adhesion sites, thereby reducing bacterial infection.^35^

Beyond simple ligand–receptor binding, researchers have developed advanced CNP formulation with a “lure-and-kill” mechanism for neutralizing phospholipase A2 (PLA2).^36^ This mechanism leverages the interactions among cell membranes, melittin, and a PLA2 inhibitor. Specifically, melittin serves as a PLA2 attractant, spontaneously integrating into the RBC membrane and attracting PLA2 to attack. Concurrently, oleyl-oxyethyl-phosphorylcholine (OOPC), a lipophilic PLA2 inhibitor, is incorporated into the membrane to neutralize PLA2 upon its attack. These CNPs have demonstrated survival benefits in animals experiencing acute PLA2 toxicity. Building on this concept, macrophage membrane-based “lure-and-kill” CNPs have also been created, which were able to inhibit PLA2 and neutralize inflammatory cytokines. When tested in an experimental model of acute pancreatitis, these CNPs effectively alleviated inflammation, reduced tissue damage, and decreased lethality associated with acute pancreatitis.^37^

Table 1 summarizes CNP formulations that neutralize targets via cell membrane. The initial design of CNPs for multiplex countermeasures primarily relied on the protein and lipid receptors present on the natural cell membranes. Due to the diversity of these natural receptors, the CNPs can neutralize a wide range of chemical and biological agents through membrane binding. Compared to other neutralization strategies, the CNP platform offers an innovative approach in developing countermeasure solutions that are driven by the functions of the target agents rather than their molecular structure.

**Table 1:** Summary of CNP Formulations That Neutralize Targets via the Cell Membrane

Membrane type	Target	Mechanism of action	Example	Reference
RBC	Pore-forming toxin	Toxin–membrane specific interaction	α-Hemolysin	(5, 14−23)
			Streptolysin O	
			Listeriolysin O	
			MRSA secreted protein	
RBC and platelet	Pathological antibody	Antibody–cell receptor binding	Anti-RBC antibody	(7)
			Antiplatelet antibody	(26)
RBC and mitochondrial outer membrane	Small molecule toxicant	Molecule–receptor binding	Organophosphate	(8)
			Anticancer compound	(28)
Macrophage	LPS	LPS–membrane receptor binding	LPS	(29)
Macrophage and neutrophil	Inflammatory cytokine	Cytokine–membrane receptor binding	IL-6	(10, 29)
			IL-1β	
			TNF-α	
T cell, macrophage, and lung epithelial cell	Virus	Virus–membrane receptor binding	HIV SARS-CoV-2	(31−33)
RBC and macrophage	Enzyme	“Lure-and-kill” mechanism	PLA2	(36, 37)
Gastric epithelial cell and bacterial outer membrane	Bacterium	Bacterium–membrane receptor binding	*H. pylori*	(34, 35)

### Concurrent Neutralization via Cell Membrane and Nanoparticle Core

In the first category of CNPs described above, the nanoparticle cores primarily stabilized the cell membrane coating while remaining inactive in neutralizing the target agents. It is hypothesized that if the cores are designed to participate in neutralizing target agents, the resulting CNP formulations would be more effective than those relying solely on the membranes. This hypothesis has led to the development of the second category of CNPs for multiplex countermeasures.^38,39^

**Figure 5:**
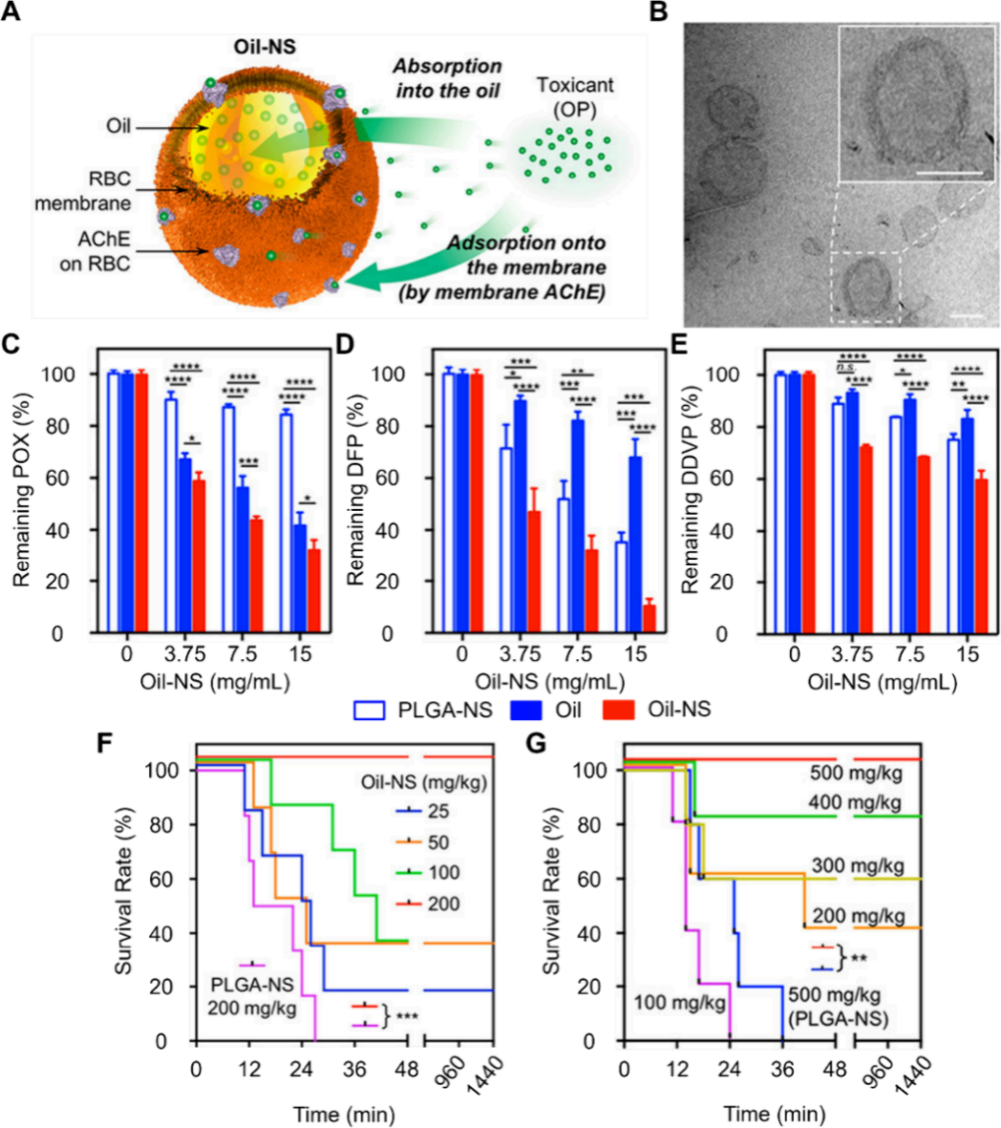
CNPs consisting of cell membrane-coated oil nanosponges (denoted as “Oil-NS” in the original study, ref (40)) were developed for dual-modal neutralization against organophosphates (OP) nerve agents. (A) Schematic depiction of Oil-NS as bimodal scavengers for OPs by simultaneously engaging the acetylcholinesterase (AChE) on the membrane for binding with OPs and the oil core to dissolute OPs. (B) TEM image illustrating the spherical core–shell structure of Oil-NS (scale bar = 100 nm). (C–E) The efficacy of Oil-NS in neutralizing paraoxon (POX, C), diisopropyl fluorophosphate (DFP, D), and dichlorvos (DDVP, E) in a dose-dependent manner. Control groups include PLGA nanoparticles coated with RBC membrane (PLGA-NS) and uncoated oil droplets. The initial OP concentration was set at 2 mg/mL for all groups. (F) In vivo treatment efficacy. Mice received a subcutaneous lethal dose of POX (0.7 mg/kg, LD100), followed by intravenous injection of Oil-NS or PLGA-NS at varied dosages. Survival rates were observed and recorded over 24 h (*n* = 6). (G) In vivo preventive efficacy. Mice were pretreated with varying dosages of Oil-NS or PLGA-NS via intraperitoneal injection. They were challenged with a subcutaneous injection of POX at a lethal dose (0.7 mg/kg, LD100) 2 min later. Survival rates were monitored for 24 h (*n* = 5). ns: not significant, *p < 0.05, **p < 0.01, ***p < 0.001, ****p < 0.0001. Adapted with permission from ref (40). Copyright 2019, the American Chemical Society.

For instance, oils have long been formulated into nanodroplets to absorb hydrophobic toxicants such as anticancer drugs and antidepressants preferentially.^41^ Based on this property, RBC membrane was coated onto oil droplets for neutralizing OPs (Oil-nanosponges or Oil-NS, Figure 5).^40^ In this construct, the cell membrane shell absorbs and neutralizes OPs through biological binding. The oil core nonspecifically soaks up OPs through the physical partition. With such a dual-modal mechanism, the Oil-NS demonstrated a higher efficacy in scavenging OPs such as paraoxon, diisopropyl fluorophosphate, and dichlorvos than their counterparts with solid polymeric cores. In mouse models of OP intoxication, Oil-NS led to higher survival rates.

**Figure 6:**
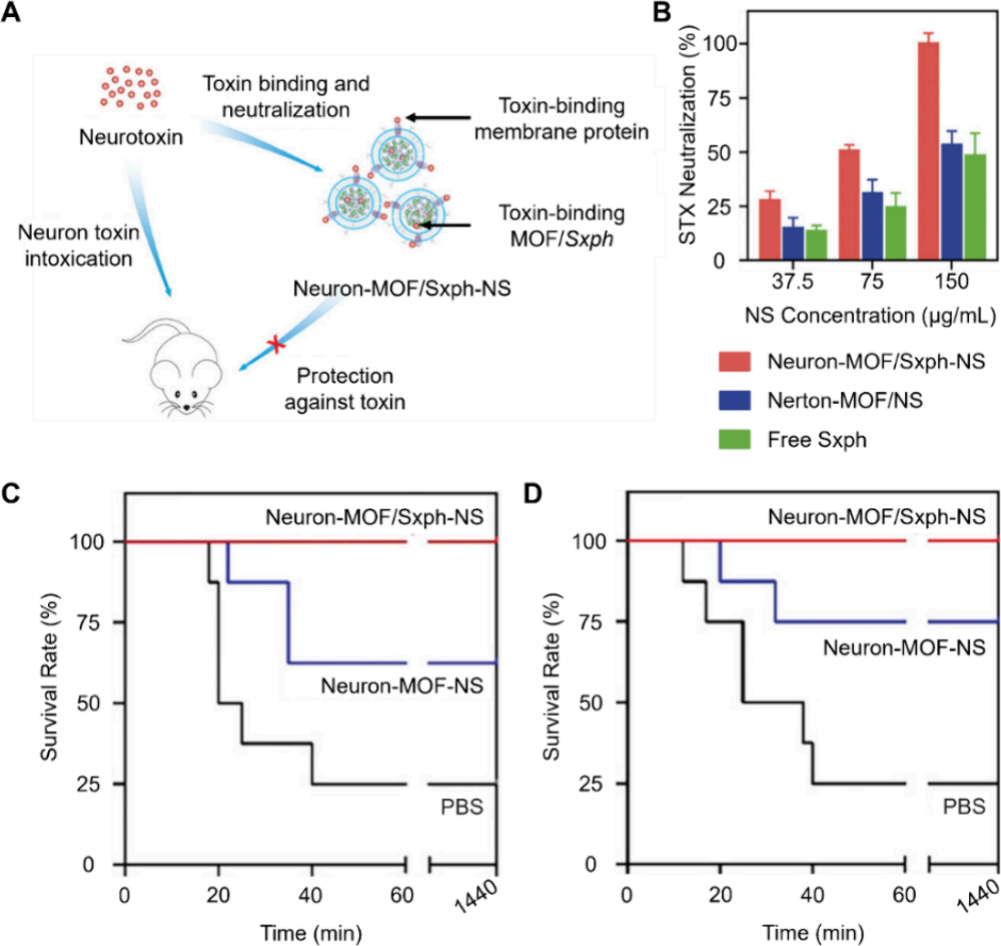
Saxiphilin (Sxph)-loaded CNPs for dual-biomimicry neurotoxin neutralization. (A) Schematic of the human neuronal membrane-coated and Sxph-loaded MOF nanosponges (denoted as “Neuron-MOF/Sxph-NS” in the original study, ref (41)). These nanosponges use a dual-biomimicry approach with a neuronal membrane coating and a MOF core encapsulating Sxph, a saxitoxin (STX)-binding protein. (B) In vitro neutralization of STX by Neuron-MOF/Sxph-NS. The sodium flux fluorescence assay assesses the percentage of STX neutralized by varying concentrations of Neuron-MOF/Sxph-NS, Neuron-MOF-NS (no Sxph payload), or free Sxph. (C) In vivo neutralization of STX by Neuron-MOF/Sxph-NS in a treatment regimen. Mice were initially administered a subcutaneous injection of STX at a dosage of 4.5 μg/kg. They received an intravenous injection of Neuron-MOF/Sxph-NS (70 mg/kg), Neuron-MOF-NS (70 mg/kg), or PBS 5 min later. The survival rates of the mice were monitored over 24 h. (D) In vivo neutralization of STX by Neuron-MOF/Sxph-NS in a preventative regimen. The mice were first given an intravenous dose of Neuron-MOF/Sxph-NS (70 mg/kg), Neuron-MOF-NS (70 mg/kg), or PBS. They were injected with STX (4.5 μg/kg) subcutaneously 5 min later. The survival rates were recorded over 24 h. Adapted with permission from ref (42). Copyright 2023, Wiley-VCH Verlag GmbH & Co. KGaA, Weinheim.

Researchers also developed CNPs with cores made from metal–organic frameworks (MOFs), which allow protein encapsulation. Unlike Oil-NS, the protein payload scavenges toxicants through specific binding instead of physical dissolution. By selecting proper binding proteins, the resulting CNPs can neutralize different toxin targets. For instance, MOF cores were coated with human neuronal membrane to counteract saxitoxin (STX), one of the deadliest neurotoxins (Figure 6).^42^ In this formulation, the neuronal membrane can bind to STX through voltage-gated sodium channels on the surface. The MOF core can encapsulate saxiphilin (Sxph), a protein that naturally binds with STX with a high affinity. The resulting CNP possesses dual neutralization mechanisms, showing higher efficacy in neutralizing STX *in vitro* than their counterpart without the Sxph payload. Additionally, it conferred a higher survival benefit in a mouse model of STX intoxication.

Table 2 summarizes CNP formulations that simultaneously neutralize targets through both cell membrane and nanoparticle core. While these CNPs can neutralize targets concurrently, their effectiveness is often constrained by issues related to solubility or binding stoichiometry.

**Table 2:** Summary of CNP Formulations That Concurrently Neutralize Targets via Cell Membrane and Nanoparticle Core

Membrane type	Core material	Synthesis method	Target	Mechanism of action	Reference
RBC	Olive oil	Membrane coating onto oil droplet via sonication	Organophosphate	Specific binding with membrane AChE receptor and nonspecific physical dissolution by nanoparticle core	(40)
Neuron	MOF encapsulating toxin-binding protein	One-pot mixing followed by extrusion	Neuron toxin	Specific binding with membrane ion channel and encapsulated toxin-binding protein	(42)

### Continuous Neutralization via Encapsulated Enzyme

In the second category of CNPs, neutralization capacity is limited once the membrane and the core are saturated by the target agents. Substituting the oil core or the binding protein with enzymes that can degrade the toxicant will likely increase the neutralization capacity by enabling continuous neutralization.

**Figure 7:**
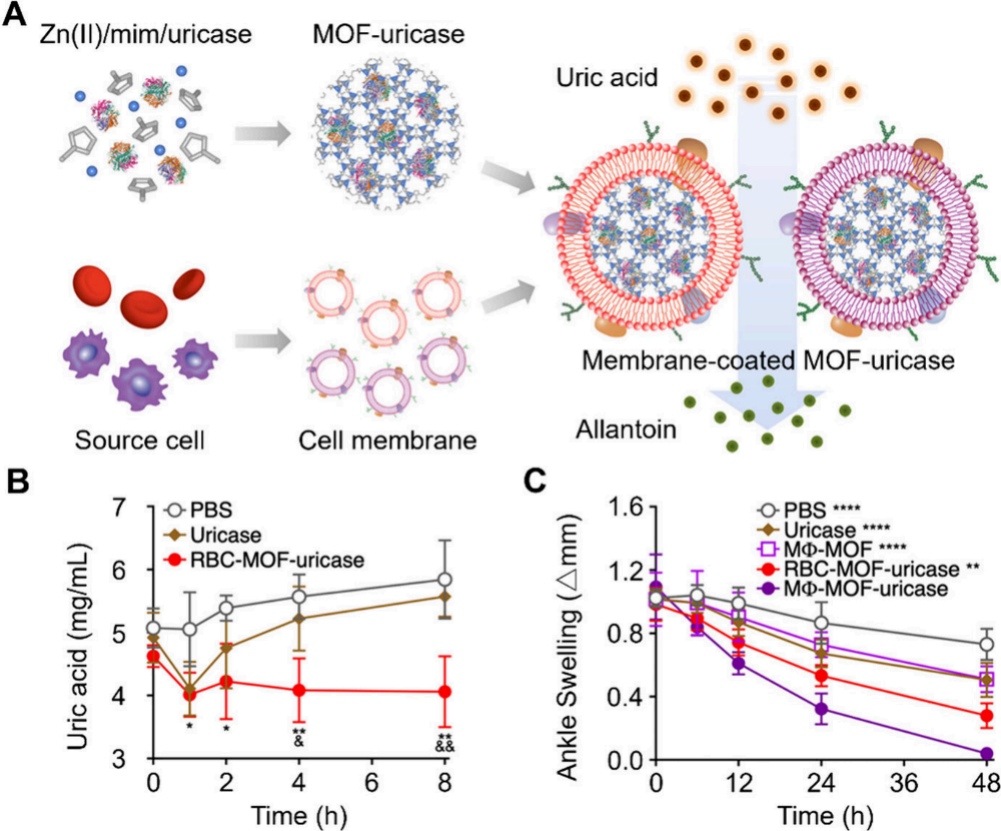
Enzymatic degradation of uric acid using cell membrane-coated uricase-loaded MOF nanoparticles. (A) Schematic representation of the synthesis of cell membrane-coated uricase-loaded MOF nanoparticles for enzymatic uric acid degradation. The process begins with forming MOF-uricase cores by incorporating the enzyme into a framework composed of Zn(II) and 2-methylimidazole (mim). These cores are then encapsulated within cell membranes derived from RBCs or MΦs. (B) In vivo management of hyperuricemia. Serum uric acid levels over time in hyperuricemic mice treated intravenously with either PBS, free uricase, or RBC-MOF-uricase. *p < 0.05, **p < 0.01 for PBS compared with RBC-MOF-uricase; ^&^*p* < 0.05, ^&&^*p* < 0.01 for free uricase compared with RBC-MOF-uricase. (C) In vivo management of gout. Changes in ankle joint diameter in gout-afflicted mice following intra-articular treatment with PBS, free uricase, MΦ-MOF, RBC-MOF-uricase, or MΦ-MOF-uricase. **p < 0.01, ****p < 0.0001 compared with MΦ-MOF-uricase at 48 h. Adapted with permission from ref (43). Copyright 2021, American Chemical Society.

The enzymes can be endogenous. For example, uricase, which converts uric acid into more excretable urea, was encapsulated in MOF cores and the cores were then coated with RBC or MΦ membranes to form uricase CNPs (Figure 7).^43^ In this study, RBC membrane-coated MOF-uricase CNPs were systemically administered to catalyze the efficient degradation of serum uric acid in hyperuricemic mice. MΦ membrane-coated MOF-uricase CNPs were administered locally into the joints of mice with gout, where the cytokine-neutralizing property of the MΦ membrane synergized with the uricase to alleviate disease symptoms. Both formulations showed superior in vivo efficacy compared to their counterparts without uricase enzyme loading.

Some enzymes for continuous degradation are inspired by nature. For example, organophosphorus hydrolase (OPH) is a bacterial enzyme that degrades various OPs. Recombinant OPH (rOPH) was encapsulated into zeolitic imidazolate framework (ZIF)-8 cores and coated with hybrid membranes made from RBC membrane and synthetic lipids. These CNPs effectively protected mice against a single lethal methyl paraoxon challenge, repeated lethal methyl paraoxon challenge, and sublethal methyl paraoxon intoxication. The hybrid membrane was blended with monosialoganglioside (GM1) for delivery. GM1 facilitated CNPs penetrating the blood-brain barriers, potentially increasing OP neutralization in the central nervous system.^44^

**Figure 8:**
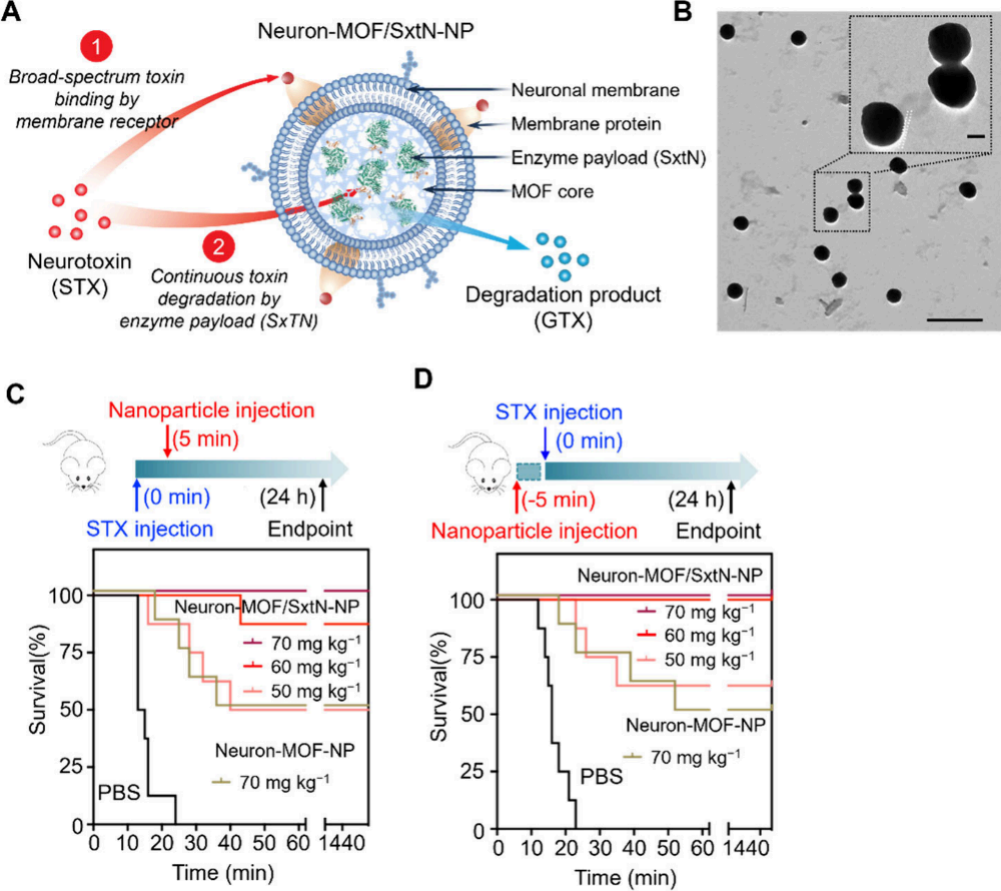
Dual-modal CNPs for continuous neurotoxin detoxification. (A) Schematic representation of neuronal membrane-coated ZIF-8 core encapsulating *N*-sulfotransferase (denoted “Neuron-MOF/SxtN-NPs” in the original study, ref (44)) illustrating dual-modal STX detoxification: STX binding by neuronal membrane receptors and STX degradation by encapsulated SxtN enzyme. (B) A representative TEM image of Neuron-MOF/SxtN-NPs (scale bar = 1 μm). Inset: a zoomed-in image of the nanoparticles showing a visible membrane shell enveloping a core (scale bar = 100 nm). (C) In a treatment regimen, survival rates of mice subcutaneously injected with 4.75 μg kg^–1^ STX, followed by intravenous administration of either Neuron-MOF/SxtN-NPs or Neuron-MOF-NPs in 5 min. (D) In a prevention regimen, survival rates of mice intravenously administered with either Neuron-MOF/SxtN-NPs or Neuron-MOF-NPs, followed by subcutaneous injection of 4.75 μg kg^–1^ STX after 5 min. Adapted with permission from ref (45). Copyright 2024 American Chemical Society.

In a recent study, researchers developed a dual-modal CNP for continuous neurotoxin neutralization. The formulation involves encapsulating the metabolic enzyme *N*-sulfotransferase (SxtN) into ZIF-8 cores and coating them with natural neuronal membrane (Neuron-MOF/SxtN-NPs) (Figure 8).^45^ The neuronal membrane possesses a high level of voltage-gated sodium channels, allowing for broad-spectrum neurotoxin neutralization. The SxtN payload enables continuous neurotoxin neutralization. The MOF core protected the SxtN payload from degradation by proteases and heat. In vitro functional assays, including a neuron osmotic swelling assay, a neuron Na^+^ flux fluorescence assay, and a cell viability assay, demonstrated the continuous and enhanced effectiveness of the formulation in neutralizing the effects of STX. Studies conducted using a mouse model of STX intoxication reveal markedly improved survival rates compared to control groups while not causing apparent acute toxicity in both treatment and prevention regimens.

Table 3 summarizes CNP formulations that achieve continuous neutralization of targets through encapsulated enzymes. These formulations use both cell membrane shell and enzymes loaded in nanoparticle core for simultaneous neutralization, addressing limitations related to solubility and stoichiometry.

**Table 3:** Summary of CNP Formulations That Continuously Neutralize Targets via Encapsulated Enzyme

Membrane type	Enzyme payload	Target	Mechanism of action	Reference
RBC and macrophage	Uricase	Uric acid	Specific degradation by uricase	(43)
RBC	Organophosphorus hydrolase (OPH)	Organophosphate	Specific degradation by OPH	(44)
Neuron	*N*-Sulfotransferase (SxtN)	Saxitoxin (SAX)	Specific binding with membrane ion channel and specific degradation by SxtN	(45)

### Enhanced Neutralization via Modified Cell Membrane

Various surface modification techniques have recently been applied to enhance the functionality of CNPs, broadening their applications in numerous research fields.^12^ These modifications have led to enhanced neutralization capacity and efficacy through multiple mechanisms.

One mechanism involves expressing higher levels of binding moieties on the cell membranes. For instance, the entry and binding of SARS-CoV-2 virus are mediated by its spike glycoprotein (S protein), which interacts with human angiotensin-converting enzyme 2 (ACE2) receptors and glycosaminoglycans such as heparin.^46^ Therefore, CNPs can mimic host cells, attracting and neutralizing SARS-CoV-2 through natural cellular receptors, offering a broad-spectrum antiviral strategy. Researchers treated THP-1 MΦs with *N*-azidoacetylmannosamine-tetraacylated (Ac4ManNAz), which metabolized into *N*-azidoacetyl neuraminic acid and incorporated into glycans for expression.^47^ The membrane was then derived and coated on PLGA nanoparticle cores. After the coating, heparin functionalized with the dibenzocyclooctyne group (DBCO-heparin) was conjugated onto N_3_-expressing nanosponges through copper-free click chemistry. This process resulted in CNPs with heparin density controlled by heparin-nanoparticle stoichiometry. CNPs with a higher heparin density showed a higher binding capacity with viral S proteins and significantly greater inhibition efficacy against SARS-CoV-2 infectivity.

**Figure 9:**
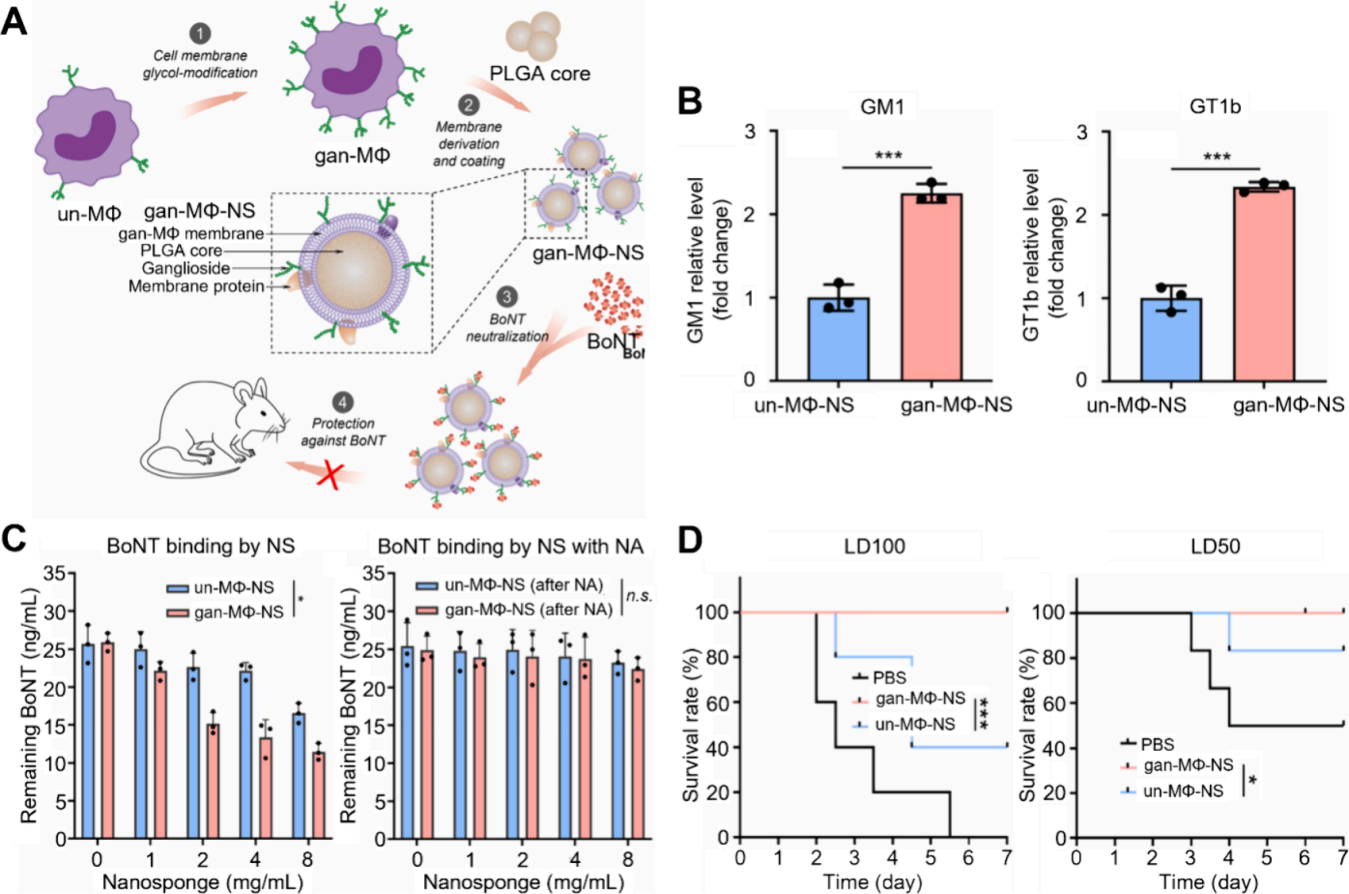
Glycan-modified CNPs for enhanced neutralization of botulinum toxin (BoNT). (A) Schematic illustration of the concept behind modifying CNPs to enhance BoNT neutralization. The study used MΦs as the source cells: un-MΦs (unmodified MΦs), gan-MΦs (MΦs with enhanced glycan expression), un-MΦ-NS (nanosponges coated with membranes of un-MΦs), and gan-MΦ-NS (nanosponges coated with membranes of gan-MΦs). (B) Comparison of GM1 and GT1b expression levels on gan-MΦ-NS and un-MΦ-NS. Gan-MΦ-NS exhibited 2.4-fold higher GM1 and 2.2-fold higher GT1b expressions compared to un-MΦ-NS. (C) The binding capacity of BoNT by nanosponges with the involvement of neuraminidase (NA). BoNT was co-incubated with nanosponges at various concentrations for 30 min, after which the remaining BoNT in the supernatant was collected via centrifugation and measured by ELISA. Gan-MΦ-NS showed accelerated binding, while pretreatment of nanosponges with NA negated the binding ability, confirming the contribution of highly expressed gangliosides to increased binding capacity. (D) In vivo survival rates over 7 days for mice exposed to BoNT at dosages of LD50 or LD100. Injection of gan-MΦ-NS at 100 mg/kg concentration successfully rescued all BoNT-challenged mice at both LD50 and LD100 doses. Adapted with permission from ref (48). Copyright 2023 Elsevier.

A similar mechanism has been utilized to develop CNPs for enhanced neutralization of botulinum toxin (BoNT), a potent neurotoxin with significant biowarfare and bioterrorism potential.^49^ Effective countermeasures against BoNT intoxication are currently lacking. Studies have shown that polysialylated gangliosides on the cell surface, such as GM1 and GT1b, are critical receptors for BoNT binding and toxicity.^50^ Researchers treated THP-1 MΦs with Ac4ManNAc, promoting ganglioside expression on the membrane via sialic acid metabolism and glycosylation (Figure 9).^48^ This treatment resulted in a 2.4-fold increase in GM1 expression and a 2.2-fold increase in GT1b expression. The modified membrane was then coated onto PLGA nanoparticle cores, producing CNPs with enriched gangliosides. These glycan-modified CNPs demonstrated higher BoNT binding capacities than CNPs made with unmodified membrane. They also showed a higher efficacy in neutralizing BoNT cytotoxicity in vitro than their unmodified counterparts. Additionally, these CNPs provided higher survival benefits in a mouse model of BoNT intoxication.

**Figure 10:**
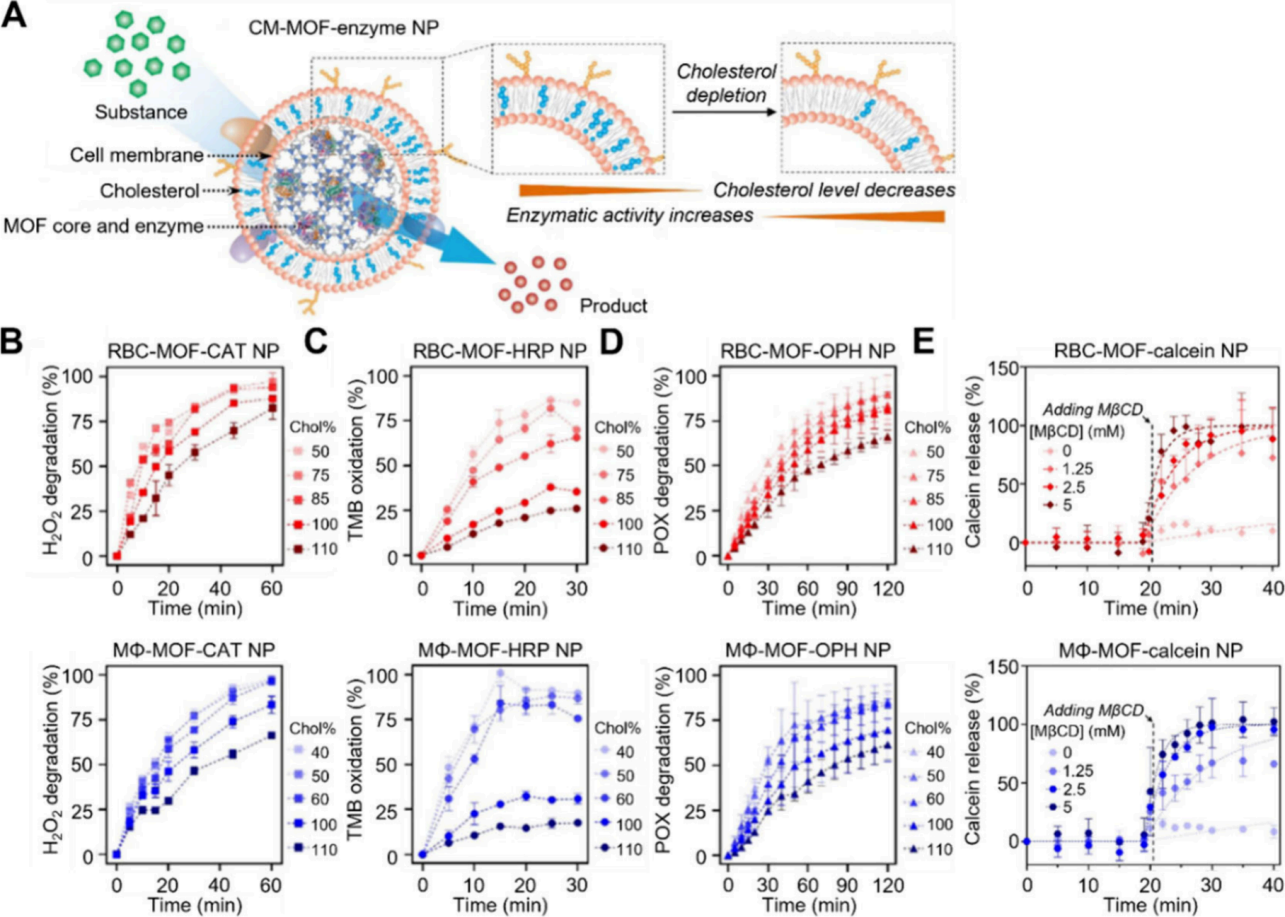
Membrane cholesterol depletion enhances the enzymatic activity of cell membrane-coated MOF nanoparticles. (A) Schematic illustration of the design of cell membrane-coated, enzyme-loaded MOF nanoparticles with reduced membrane cholesterol content to enhance enzymatic activity. Three enzymes were tested: catalase (CAT), horseradish peroxidase (HRP), and organophosphorus hydrolase (OPH). (B–D) The percentage of H_2_O_2_ (B), 3,3′,5,5′-tetramethylbenzidine (TMB) (C), and paraoxon (POX) (D) degradation over time after incubation with RBC-MOF-CAT NPs or MΦ-MOF-CAT NPs with varying membrane cholesterol levels. Reduced membrane cholesterol enhances substrate degradation. (E) The calcein release from the nanoparticles correlates with the amounts of methyl-β-cyclodextrin (MβCD) applied to deplete membrane cholesterol. Adapted with permission from ref (51). Copyright 2022 John Wiley & Sons, Inc.

The reactivity of enzyme-loaded CNPs depends on the rate at which substrates diffuse across the membrane into the cores and how quickly products diffuse out once generated. Cholesterol plays a crucial role in modulating membrane permeability.^52^ The dense packing of phospholipids induced by cholesterol reduces membrane permeability, hindering molecular transport. Conversely, cholesterol depletion increases membrane permeability to small molecules. Therefore, enhancing the efficiency of continuous neutralization by depleting membrane cholesterol is another promising mechanism. Researchers demonstrated this mechanism by encapsulating catalase, horseradish peroxidase, and organophosphate hydrolase into ZIF-8 CNPs and coating them with human RBC or MΦ membranes (Figure 10).^51^ Reducing cholesterol levels enhanced enzymatic activity considerably, suggesting the potential to tailor CNP neutralization efficiency by adjusting membrane cholesterol levels.

Another intriguing mechanism involves cell membrane modification to achieve higher stealth and prolonged residence time of CNPs, thereby enhancing their neutralization effectiveness. For instance, researchers engineered MΦs to express proline-alanine-serine (PAS) peptide chains, which offered additional protection against opsonization and phagocytosis besides native membrane moieties.^53^ The modified CNPs exhibited prolonged residence times when administered intravenously or intratracheally, surpassing those coated with the wild-type membrane, and the extended residence time led to increased efficacy in inhibiting inflammatory cytokines in mouse models of LPS-induced lung injury and sublethal endotoxemia. The versatility of this technique is inspiring, as it could be extended to other membrane types for more favorable biomedical applications.

Dual-membrane hybrid CNPs, which harness the synergistic bioneutralization capabilities of two distinct membranes, have been developed to improve neutralization outcomes. Unlike single-membrane CNPs, these hybrid CNPs integrate the biofunctions of natural protein receptors from different cell membranes, achieving a synergy that is otherwise difficult to achieve. A notable example is CNPs coated with a hybrid membrane of RBCs and platelets.^54^ In this design, the platelet membrane targets and neutralizes bacteria through interactions between platelet von Willebrand factor and fibrinogen with bacterial clumping factor A (clfA). The RBC membrane enhances the efficiency of toxin neutralization. This hybrid membrane was applied to ultrasound-propelled, fuel-free gold nanorobots (RBC-PL robots). These nanorobots, driven by ultrasound, effectively neutralized pore-forming toxins from MRSA bacteria. Additionally, the RBC-PL robots demonstrated superior performance in isolating MRSA USA300 strain bacteria, known for high platelet adhesion. The ultrasound-propelled RBC-PL robots also caused less hemolysis than static RBC-PL robots (17% versus 40%) and exhibited anti-biofouling properties, allowing them to move through whole blood without losing speed.

Table 4 summarizes CNP formulations with enhanced neutralization capabilities achieved through modified cell membranes. These designs focus on adding extra ligands to boost binding capacity or improving cell membrane permeability to enhance enzymatic degradation. As cell membrane functionalization techniques advance, we expect the emergence of more refined strategies tailored to specific targets, resulting in greater neutralization effectiveness.

**Table 4:** Summary of CNP Formulations That Have Enhanced Neutralization Capability via Modified Cell Membrane

Membrane type	Modification method	Target	Mechanism of action	Reference
Macrophage	Conjugation of heparin onto membrane via click chemistry	SARS-CoV-2	Virus–membrane receptor binding	(47)
Macrophage	GM1 and GT1b overexpression via metabolic engineering	Botulinum toxin (BoNT)	Enhanced binding of BoNT to cell membrane	(48)
RBC and macrophage	Depletion of cell membrane cholesterol	H_2_O_2_	Enhanced H_2_O_2_ permeation and specific degradation by catalase	(51)
		3,3′,5,5′-Tetramethylbenzidine (TMB)	Enhanced TMB permeation and specific degradation by horseradish peroxidase	
		Paraoxon (POX)	Enhanced POX permeation and specific degradation by OPH	

## Conclusions and Outlook

In summary, CNPs stand out in nanomedicine for their ability to mimic source cells, leading to rapidly expanding applications across various research areas. This review focuses on their role in multiplex neutralization, where CNPs emulate host cells to act as alternative targets, intercepting harmful agents. Unlike traditional methods, CNPs neutralize target agents based on their function rather than structure, enabling broad-spectrum neutralization. CNP designs and mechanisms have significantly evolved since the inception of the concept over a decade ago. This review traces this evolution, summarizing the platform’s development four categories with progressive functions: neutralization via cell membrane binding, concurrent neutralization via cell membrane and nanoparticle core, continuous neutralization via encapsulated enzyme, and enhanced neutralization via modified cell membrane. Key examples of each category are analyzed, emphasizing the structure–function relationship of CNPs. From an engineering perspective, we also summarize the principles of their synthesis (Table 5). The review provides insights into the progress of CNP technology, aiming to inspire additional applications and advancements for future development. Although this article discusses four categories of CNP designs, the technologies within each category can be integrated to create powerful CNP formulations for multiplex countermeasures.

**Table 5:** Summary of Synthesis Principles of Major CNPs Reviewed in This Article

Construct feature	Synthesis principle	Reference
CNPs with cell membrane and solid core (without payload)	Purified cell membrane is coated onto nanoparticle core via sonication or extrusion	(5), (29)
CNPs with cell membrane and oil core	Oil droplets are added to membrane suspension, followed by sonication	(40)
CNPs with cell membrane and MOF core (with enzyme payload)	MOF building blocks and enzyme payload are mixed with membrane vesicles, followed by extrusion	(43), (51)
CNPs coated with a hybrid membrane	Different cell membranes are combined and sonicated for hybridization, and the resulting hybrid membrane is then used to formulate CNPs	(54)
Lure-and-kill CNPs	Lipid-like PLA2 inhibitors are mixed with cell membrane and sonicated, and the resulting membrane is then used for coating, followed by doping with melittin	(37)

The unmatched advantages of using CNPs for multiplex neutralization have sparked curiosity about the downstream translation of this platform, prompting the identification of several critical considerations. The combination of natural and synthetic materials in these biomimetic nanoparticles poses scalability, manufacturing, and regulatory challenges. While established protocols and infrastructure exist for collecting and banking blood cells like RBCs, platelets, and leukocytes, large-scale cell culture adaptation will be imperative for other cell types. Developing high-capacity and high-yield methods for cell membrane derivation and coating onto substrates is also paramount. Additionally, effective assays will be indispensable for evaluating CNP purity and stability. Given the complexity of cell products, adherence to good manufacturing practices to ensure elevated purity and consistent quality is critical.

Another major challenge is to address the uptake, distribution, clearance, and toxicity of nanoparticles, especially given the increasing diversity of materials and the complex nature of nanoparticle formulations. These aspects often require case-by-case studies to fully understand their implications. For instance, as research into MOFs has expanded rapidly, a broader variety of MOF nanoparticles have been investigated. Studies have increasingly elucidated the correlations between their in vivo clearance and factors such as chemical composition, size, surface properties, and morphology. These advancements have led to the development of low-toxicity MOF constructs, potentially benefiting MOF-based CNP development.^55,56^ As another example, hybrid CNPs combining multiple targeting mechanisms has led to enhanced targeting capability.^57^ The strategy also helps to mitigate the short circulation lifetime associated with more immunogenic membranes. Although the benefits of hybridization were demonstrated in cancer treatment, the strategy is expected to be applicable for biological neutralization applications. However, despite the promise, as a therapeutic modality distinct from the traditional small molecule drugs, antibodies, and cell therapies, the CNP-based drug product candidates present regulatory challenges that require close communications with regulatory agencies and their guidance. These challenges are similarly present in other CNP applications, such as drug delivery and nanovaccines.^58,59^ Despite these hurdles, the significant potential and benefits of CNP platforms promise tremendous opportunities to advance nanomedicine in the foreseeable future.

## VOCABULARY

Cellular Nanoparticles: These are nanoparticles designed to mimic cells by coating synthetic cores with natural cell membranes, which can be either unmodified or genetically engineered. This cell membrane coating gives the nanoparticles unique properties that make them effective for drug delivery, detoxification, and various other biomedical applications.
Cellular Nanosponges: These are cellular nanoparticles specifically designed to neutralize harmful chemical and biological agents, including chemical toxicants, biological toxins, cytokines, and pathogens.
Detoxification: It is the process of removing toxic substances or neutralizing their harmful effects within a living organism.
Multiplex Countermeasure: A strategy or intervention that can simultaneously address or neutralize various threats, such as chemical agents, toxins, cytokines, or pathogens. This approach enhances the effectiveness and scope of protection by targeting multiple harmful factors at once.
Continuous Neutralization: A process or mechanism that consistently and actively neutralizes harmful substances, such as chemical agents, toxins, or pathogens, over an extended period. This continuous action helps maintain a safe environment by preventing the accumulation of harmful effects of these substances.
